# The Effects of *Plasmodium vivax* Gestational Malaria on the Clinical and Immune Status of Pregnant Women in Northwestern Colombia

**Published:** 2013-09-30

**Authors:** María Fernanda Yasnot, Douglas Jay Perkins, Mauricio Corredor, Stephanie Yanow, Jaime Carmona-Fonseca, Amanda Maestre

**Affiliations:** 1 Escuela de Ciencias Basicas, Facultad de Ciencias de la Salud, Universidad del Valle, Cali, Colombia and Grupo de Investigaciones Microbiologicas y Biomedicas de Cordoba, Universidad de Cordoba, Montería, Colombia.; 2 University of New Mexico, Albuquerque, New Mexico, USA. Director, Center for Global Health.; 3 Facultad de Ciencias Exactas, Gebiomic, Universidad de Antioquia, Medellín, Colombia.; 4 Alberta Provincial Laboratory for Public Health, Edmonton, Canada.; 5 Grupo Salud y Comunidad, Facultad de Medicina, Universidad de Antioquia, Medellin, Colombia.

**Keywords:** *Plasmodium vivax*, malaria, pregnancy, placenta, cytokine, chemokine, Colombia

## Abstract

**Objetive:**

The study explored the effects of *Plasmodium vivax* infection on the balance of pro- versus anti- inflammatory cytokines and chemokines and their relationship with some clinical and epidemiology outcomes.

**Methods:**

Thirty-five pregnant women were recruited. Of these, 15 subjects had malaria at delivery (GM+), and 20 had no exposition to infection throughout the pregnancy (GM-) and at delivery. Epidemiological and clinical data were recorded after reviewing the clinical records. At delivery, whole blood from the mother as well as placental tissue was collected. Diagnosis of infection was performed by thick smear and a polymerase chain reaction (PCR). Expression of pro-inflammatory and anti-inflammatory cytokines and chemokines was measured by a real time PCR.

**Results:**

The clinical and epidemiological variables explored were similar in both groups, with the exception of gestational age. When comparing the GM+ group with the GM- group, it is clear that although the differences generally are not significant, pro- inflammatory cytokines are elevated in both maternal blood and placental; anti-inflammatory ones are elevated in the mother and reduced in the placenta, and the chemokines are reduced in both compartments, except for MCP-1 which is elevated in all.

**Conclusion:**

The results appear to be strongly affected by the small number of women with GM by *P. vivax* at childbirth. Additional studies are needed with larger groups in this and other regions of the country

## Abstract (es)

**Objetivo:**

En este estudio se determinó el efecto de la infección por *Plasmodium vivax* en el balance de citoquinas pro-inflamatorias/anti-inflamatorias y quemoquinas y su relación con algunas variables epidemiológicas y clínicas.

**Métodos:**

Se reclutaron 35 gestantes, 15 con malaria en el momento del parto (GM+) y 20 sin malaria en ningún momento de la gestación (GM-) Los datos epidemiológicos y clínicos fueron colectados a partir de la historia clínica. En el momento del parto fueron tomadas muestras de sangre periférica materna y tejido placentario. El diagnóstico fue realizado mediante gota gruesa y reaccion en cadena de la polimerasa (PCR). La expresión de citoquinas pro-inflamatorias/anti-inflamatorias y quimioquinas, fueron medidas por PCR en tiempo real. La expresión de citoquinas pro-inflamatorias/anti-inflamatorias y quemoquinas, fueron medidas por PCR en tiempo real.

**Resultados:**

En las variables epidemiológicas y clínicas, los datos fueron similares en ambos grupos. Al comparar el grupo GM+ con el grupo GM-, resulta claro que, aunque las diferencias, en general, no son significativas, las citoquinas proinflamatorias están elevadas tanto en sangre materna como placentaria, las antiinflamatorias están elevadas en la madre y reducidas en la placenta, y las quimioquinas están reducidas en ambos compartimentos, excepto la MCP-1 que está elevada en ambos.

**Conclusión:**

Los resultados parecen estar fuertemente afectados por la cantidad pequeña de mujeres con MG por *P. vivax *en el parto.

Es necesario adelantar estudios adicionales con más mujeres tanto en esta región como en otros lugares.

## Introduction

Pregnancy involves a greater risk of malaria^1^. The prevalence and severity of gestational malaria (GM) that occurs during pregnancy has been studied in Africa, Latin America, Oceania and Asia^2^. What is known about GM and placental malaria (PM) relates to *Plasmodium falciparum* and very little is known about the role of *P. vivax* ^3^. Gestational malaria patients usually present with anemia^4^. A review of seven Latin American studies on GM confirms the clinical effects of the infection with *P. falciparum* as the main causing species^5^. 

During a normal pregnancy the immune response undergoes specific changes to allow successful implanting and development of the fetus. Therefore, a Th2 profile is observed in successful pregnancies, while presence of a Th1 cytokine (pro- inflammatory) profile results in spontaneous abortion due to direct cytotoxic effects on the trophoblast. *Plasmodium* infection in the mother changes the protective cytokines balance and increases the Th1 responses leading to complications such as anemia, abortion, stillbirth, low birth weight, among other. 

The normal anti-inflammatory status observed in the uninfected placenta is mediated by the increased production of cytokines, such as interleukin (IL)-10, and transforming growth factor β (TGFβ) which probably suppress the immune response mediated by cells. During GM-PM, this type of response is required to control symptoms, while the parasite is cleared by other mechanisms ^6^.

The most studied cytokines in the physiopathology of *P. falciparum* GM are tumor necrosis factor (TNF), gamma interferon (IFNγ) and IL- 10, but other cytokines and chemokines have also been explored^7^. Many cytokines changes during GM-PM might be involved in the pathogenesis of gestational malaria resulting in low birth weight, intrauterine growth restriction, and preterm delivery.

During placental infection, increased production of TNF and IFNγ results in injury of the villous trophoblast^8^. Furthermore, macrophage inflammatory protein 1-beta (MIP-1β), TNF and IL-8 have been associated with low birth weight and intrauterine growth restriction; and Interleukin 1β, IL-6, IL-8 and TNF have all been involved in pre-term delivery^9^ ^,^ ^10^. 

Accumulation of inflammatory cells, particularly macrophages and monocytes, in the inter-villous space, results after increased MIP-1β. Consequently, both the trophoblastic morphology and placental cytokine production are affected ^11^. About 25% of women with GM have placental monocyte infiltrates at the time of delivery and this has been identified as a key factor and predictor of low birth weight. It has been suggested that infiltrating monocytes represent a significant source of pro-inflammatory cytokines in the infected placenta^12^.

Currently, no reports have been made on the immune balance during GM-PM by *P. vivax*, a species highly endemic in Asia and America. The aim of this research was to describe the expression profile of the pro-inflammatory cytokines TNF, IFNγ, IL-1β, the anti-inflammatory cytokines IL-10, IL-6, TGF-β and the chemokines IL-8 (CXCL8), MIP-1 β (CCL4) and MCP-1 (CCL2), in pregnant women and placentas infected with *P. vivax* at the time of delivery as well as to describe the association with clinical and epidemiological characteristics.

##  Materials and methods 

### Study site

The study was carried out in north-west Colombia in the locality of Puerto Libertador (Cordoba department). Between Antioquia and Cordoba departments lays the malaria endemic region known as Uraba-Altos Sinu/San Jorge-Bajo Cauca, which has an estimated area of 43,506 km2 and a malaria at-risk population of 2.5 million distributed in 35 municipalities. 

This large region generates over 60% of malarial cases in Colombia: 18% in Córdoba and 42% in Antioquia. *P. vivax* predominates over *P. falciparum* malaria in approximately a 2:1 ratio^13^. In 2006-2007, Antioquia and Córdoba contributed, on average, 61,458 cases of malaria, or 60.2% of the total for Colombia. Those two departments during the same years contributed 72.1% of the cases of *P. vivax*, 34.4% of the cases of *P. falcpiarum*, and 45.7% of the cases by both especies^14^.

Subjects for the current study were recruited at the local hospital and the antenatal clinic of "CAMU Divino Niño", in Puerto Libertador.

### Study type, design and sample size

Volunteers were enrolled as part of a descriptive, prospective and transversal study, between June 2010 and June 2012. The sample size was defined by convenience and the lengthy period of recruitment was the result of severe and prolonged political turmoil in the region, which also jeopardized a random inclusion of subjects. Therefore, recruitment took place in a sequential fashion until the sample size for the study was reached.

Subjects were grouped as follows: a) parturient women with *P. vivax* GM at delivery (GM+); b) healthy pregnant women without GM during pregnancy and at childbirth (GM-). Recruitment took place regardless on the presence clinical signs of malaria infection at the time of diagnosis.

Thirty-five pregnant participants were enrolled: 15 with GM at delivery (GM+), and 20 without GM at the antenatal clinic or at delivery (GM-).

### Criteria for inclusion and exclusion

Inclusion criteria were>1 year residency in the study region, general good health and no history of serious disease, signature of the voluntary consent form, hospital delivery and attendance to at least three antenatal check-ups. Exclusion criteria were withdrawal of the voluntary consent and the finding of a PCR positive for *P. falciparum* infection.

### Collection of clinical data

Clinical data were collected after review of the antenatal clinical records and delivery charts. Information on mother´s age, weeks of gestation, parity, mother´s hemoglobin and newborn weight, was recorded.

### Sample collection and preparation

Whole blood samples from mother´s peripheral blood and placenta were obtained at delivery rooms in the local hospitals of Puerto Libertador. The mother`s sample was obtained by finger prick and placental blood was obtained from the lake formed following a wash with saline (0.9%) and removal of a 3x3x3 cm section in the area of the cord's insertion on the maternal side of the organ. These samples were used to perform thick and thin smear examination and PCR as detailed below. In addition, 4ml of maternal peripheral blood were collected and immediately stored in liquid nitrogen, this and a RNA later^(r)^ preserved tissue fragment obtained from the placenta, were processed for expression of cytokines and chemokines.

### Diagnosis of Plasmodium infection

The presence of *Plasmodium vivax* was confirmed in maternal and placental blood by thick smear and a nested polymerase chain reaction (PCR) according to standard published methods^15^ ^,^ ^16^. Diagnosis by microscopy was carried out in the field site by a trained technician with certified experience in malaria diagnosis. Parasitaemia was calculated after observation of fields corresponding to 200 leukocytes and a constant of 8,000 leucocytes/μL. A sample was considered negative after observation of 500 high power fields.

Diagnosis of infection by PCR was performed to whole blood samples collected onto Whatman 3MM filter paper, from which DNA was extracted using Chelex100^(r)^ (Sigma(tm)). Amplification was carried out using a nested PCR assay to detect the 18 s rRNA gene of *P. vivax*, according to previously published procedures^16^. The positive reaction control consisted of DNA from a well characterized field isolate of *P. vivax*. Amplification products were resolved in a 2% agarose gel using ethidium bromide and visualized under UV light. Samples were processed once as long as controls were confirmed to operate in optimal conditions, otherwise, the assay was repeated until successful performance of controls.

For the GM+ group, at least one positive test was required for allocation and all tests were required negative for the GMC- group.

### Term definitions

The following definitions were applied: A) Low birth weight: <2,500 grams. B) Preterm delivery: a pregnancy concluded before 37 weeks. F) Maternal anaemia: haemoglobin <11 g/dL, G) Full term pregnancy: >38 weeks. H) Gestational or pregnancy malaria: plasmodial infection diagnosed in a pregnant woman after examination of maternal peripheral blood. I) Placental malaria: plasmodial infection diagnosed in placenta after examination of placental blood.

### Cytokines and chemokines expression analysis

Relative quantitation for expression analysis was performed using a reverse-transcription real time PCR assay (RT-PCR). Total RNA was extracted using QIAamp RNA Blood Mini^(r)^ (QIAGEN) and cDNA was synthesized using First Strand cDNA Synthesis^(r)^ (Fermentas). The quality of the cDNA was confirmed by using a conventional PCR for the glyceraldehyde 3-phosphate dehydrogenase (GAPDH) gene. The reaction was set up in an Applied Biosystems Step One Plus system using TaqMan^(r)^. Results were read at 530 nm. the following genes were analyzed for expression: TNF, IFNγ, IL-1β , IL-10, IL-6, TGF-β, IL-8, MIP-1 and MCP-1 . The efficiency of the PCR reactions was determined based on mRNA extracted from a stimulated BeWo cell culture or peripheral mononuclear cells from a donor. cDNA was serially diluted and expression of β-actin was used to normalize the assays using the delta delta CT method (ΔΔCt). The reaction was performed in a final volume of 10 uL containing 1 uL DNA, 5ul TaqMan(r) master mix (Applied Biosystems), and 0.5 uL of 20X TaqMan assay mix (primers and probes for each cytokine/chemokine). All primers were supplied by Applied Biosystems. Reactions were performed at Laboratory of Tropical Diseases of the University of New Mexico, USA.

### Statistical analyses

Data were analyzed using SPSS 10.0 (SPSS IBM, Armonk, NY). Proportions and medians were obtained. Significance was established at p<0.05%. The analysis included comparisons with using Mann-Whitney U test. Bivariate linear correlations were analyzed using Spearman´s rho coefficient.

### Ethical issues

The study protocol was reviewed and approved by Ethics Committee of Instituto de Investigaciones Medicas, Universidad de Antioquia (acta 012: project Colciencias code 111549326134, contract 611 de 2009). Volunteers were recruited in accordance with national guidelines (Resolution No. 008430 of October 4, 1993, Republic of Colombia, Ministry of Health), as well as international guidelines (Declaration of Helsinki and its amendments, World Medical Association (WMA), Edinburgh, Scotland, October 2000). Subjects were assigned a code which was known only by the researchers. 

## Results

### Characteristics of the population studied.

A total 35 pregnant women were included in the study, 15 were allocated to the GM+ group and 20 to the GM-. . No significant differences were observed in the clinical and epidemiological variables between the two groups (Table 1). In both groups, there was a significant linear correlation between age and parity (rho = 0.868, p = 0.000) and parity and birth weight (rho = 0.628, p = 0.000). Pre-term delivery was observed in the GM+ group and absent in the GM- group.

**Table 1:**
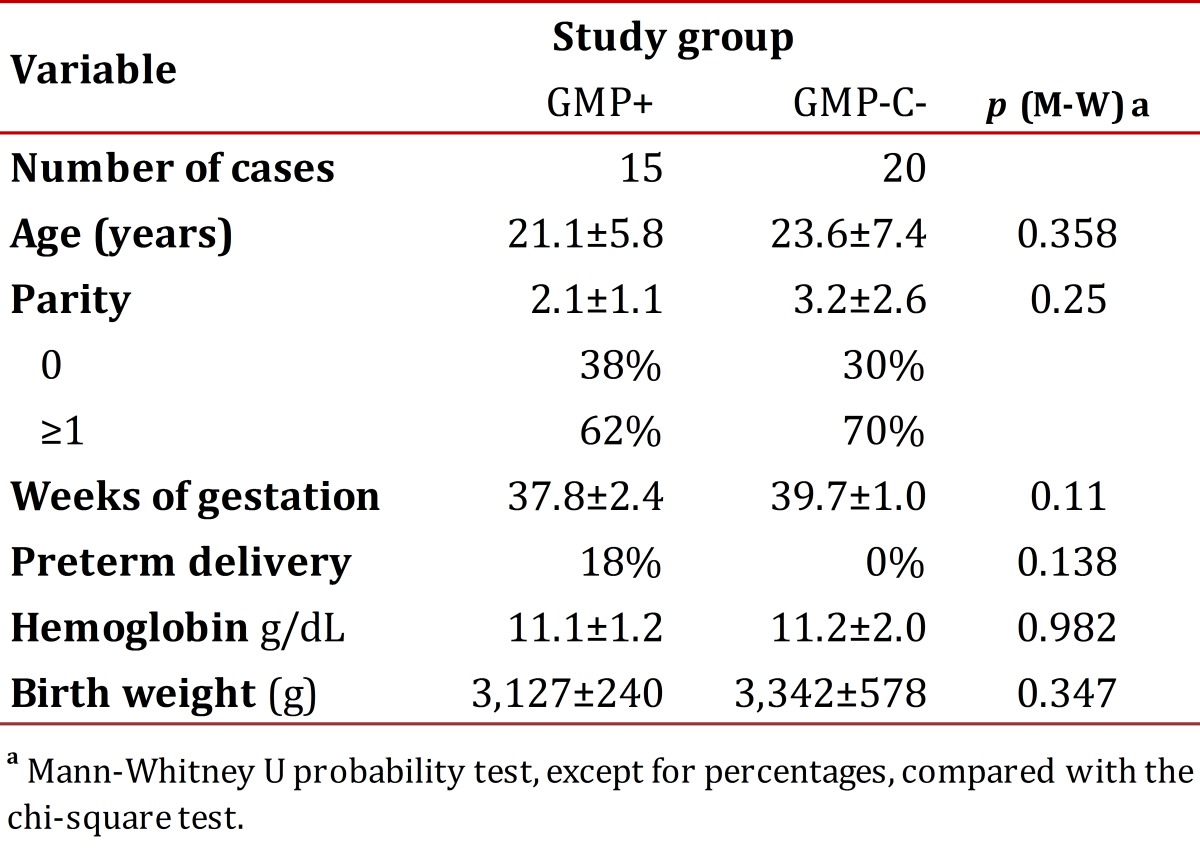
Characteristics of the study groups with and without gestational malaria

### Expression of pro-inflamatory/anti-inflamatory cytokines and chemokines in placental tissue and maternal peripheral blood.

When comparing the expression of pro-inflammatory and anti-inflammatory cytokines in placental tissue in the GM+ and GM- groups, an increase in the levels of pro-inflammatory cytokines (TNF, IFNg and IL1β) was observed in the former. Significance in this finding was confirmed for TNF. Regarding the anti-inflammatory cytokines, these were lower in the GM+ group *versus* the GM- group; however, no significance could be confirmed in this result. Similarly, the chemokine MCP-1 was observed high in the GM+, but no significant difference was confirmed (Table 2).

**Table 2:**
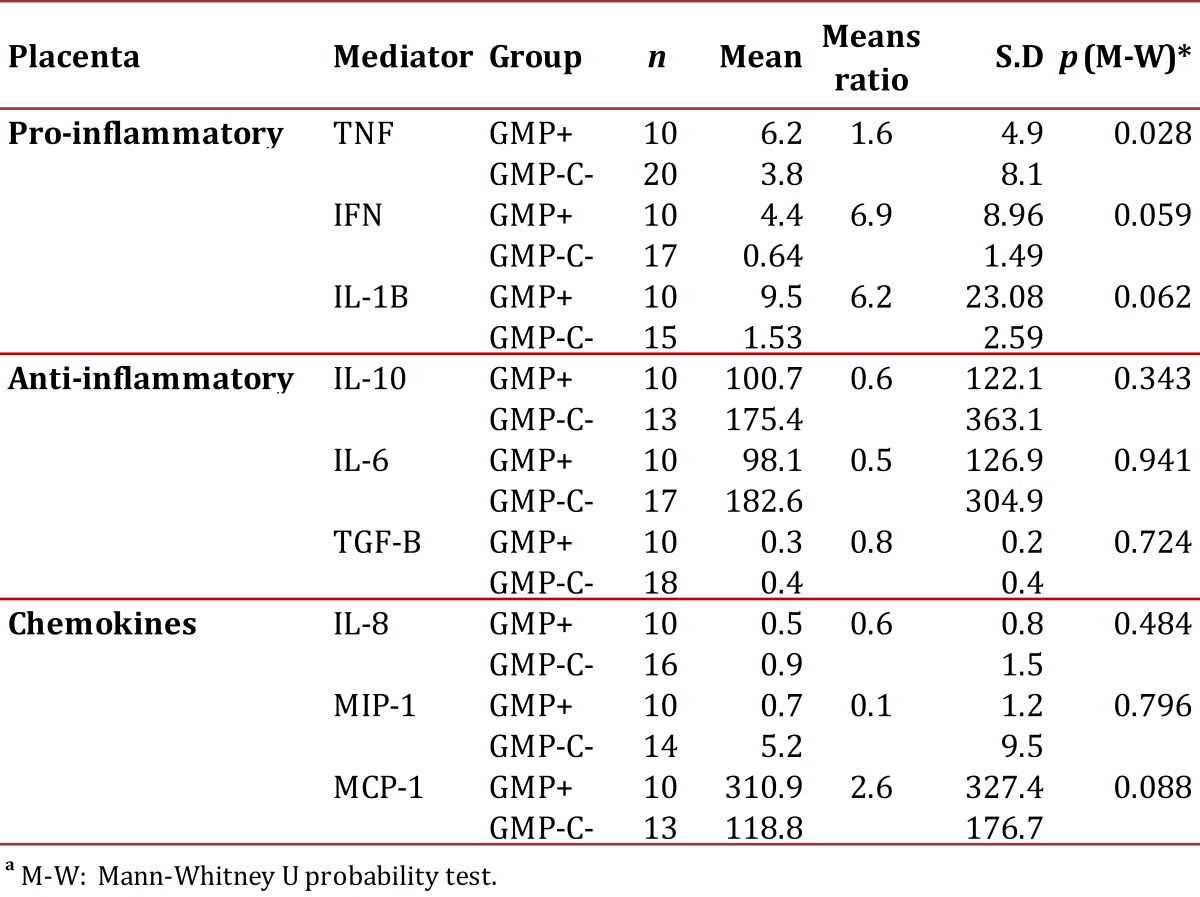
Levels of cytokine and chemokine expression in the placental tissue of pregnant women with malaria by P. vivax at delivery (P+) and without it at delivery or during prenatal care (PC-).

In maternal blood, all changes were not statistically significant. However, pro-inflammatory cytokines (TNF, IFNg and IL-1β) were higher in the GM+ group with respect to the GM- group. In addition, all anti-inflammatory cytokines, but TGF-β, were increased in the GM+ group when compared to GM-. As for the chemokines, IL-8 and MIP-1 were lower, and MCP-1 higher, in the GM+ group when compared to GM-.

**Table 3:**
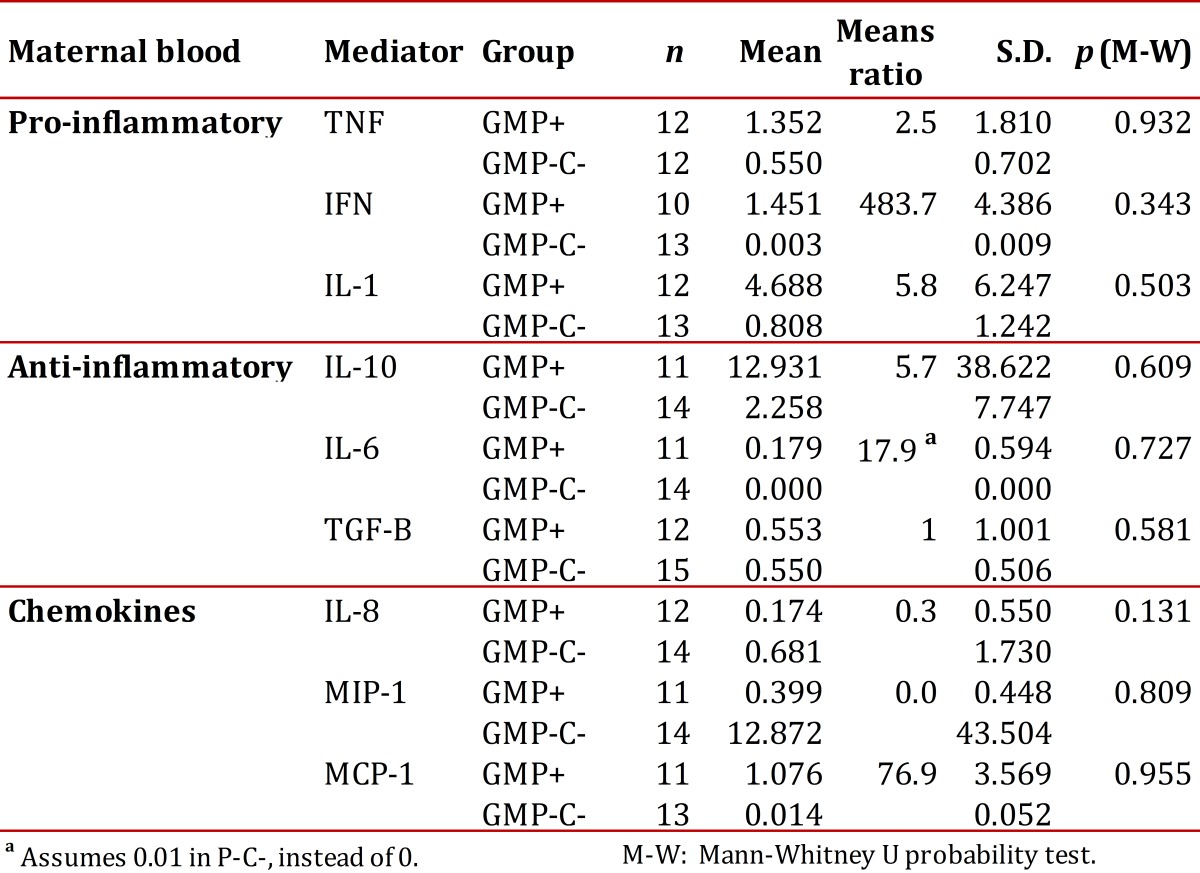
Levels of cytokine and chemokine expression in maternal peripheral blood of pregnant women with malaria by P. vivax at childbirth (GMP+) and without it at birth or during prenatal care (GMP-C-).

## Discussion

This reports for the first time on the status of cytokines and chemokines in women with GM in a *P. vivax* endemic region of America. The technique used to measure the expression of immune mediators is precise and sensitive. Volunteers were selected from the general population, particularly those regularly attending the hospital facilities in Puerto Libertador. Therefore, the study represents the typical population which seeks health services in the region.

An obvious limitation in this work is the reduced number of women with GM, which is the result of the current socio-political instability in the area. Despite the small sample size, the results obtained are consistent and suggest a clear trend. 

The study contributes to outline the epidemiology of malarial infection during pregnancy in a highly endemic region of the country, since it includes subjects whose peripheral and placental blood was monitored for *P. vivax* infection using standard microscopy and contributes with the evidence, so far gathered, on the deleterious effects of this species ^17^.

Other authors have reported on the particular predisposition to acquire malaria, regardless of the species, according to mother´s age, parity and gestational age ^18^. In the current study, women in the GM+ group were younger, with less parity and fewer weeks of pregnancy than those in the GM- group.

Others have also reported on *P. vivax* as responsible of low birth weight, maternal anemia and preterm delivery^17^. In this study, no significant differences were observed on mother´s haemoglobin levels or the newborn´s weight. However, children born from mothers with GM+ had a mean 215g less weight. In addition, preterm delivery occurred in high proportion (18%) in the same group. Preterm delivery is a recognized cause of neonatal mortality ^9^ ^,^ ^19^.

Pro-inflammatory and anti-inflammatory cytokines and chemokines were found altered during GM by *P. falciparum* and this was associated with abortion, stillbirth, preterm delivery, intrauterine growth restriction, low birth weight and maternal anemia^4^ ^,^ ^20^. However, for *P. vivax* there is no report on this matter. We found an increased expression of all pro-inflammatory cytokines studied, both in maternal peripheral blood and in placental tissue. Although differences between the groups were not significant, the probability values ​​observed strongly suggest that the sample size might have influenced this outcome. Similar to the findings herein reported, studies with *P. falciparum* GM also showed increased expression of TNF and IL1β but not IFNγ ^21^ ^,^ ^22^_. _In other reports on *P. falciparum* GM, increased TNF has been associated with fetal growth restriction, spontaneous abortion and maternal anemia ^22^ ^,^ ^23^. Likewise, the increase in IL-10 has been associated with preterm delivery and increased IFNγ with spontaneous abortion.

The beta chemokine MCP-1 has also been reported to increase in GM and PM by *P. falciparum* ^24^ ^,^ ^25^ and it has been associated with immune cell recruitment in the placenta, specifically monocyte. This chemokine might also be involved in mononuclear recruitment in *P. vivax* infected placentas. Moreover, a rise on IL-8 has been reported in placentas with active *P. falciparum *infection ^10^ ^,^ ^26^. In the current study, monocytes-macrophages infiltrates (data not shown) were observed in infected placentas and the concomitant finding of high IL-8 might explain this observation. Alternatively, other have implicated hemozoin or parasite´s persistence in the immune alterations observed in the placenta during malaria infection ^27^.

In summary, in women with GM at delivery pro-inflammatory cytokines tend to be elevated both in maternal blood and placental tissue, anti-inflammatory cytokines were high in the mother and low in the placenta and the chemokines were reduced in both compartments, but MCP-1 which was observed high in placenta and mother. The results seemed to be strongly affected by the relative small number of women with GM by *P. vivax* at the time of delivery. To consolidate these findings it is necessary to carry out additional studies with more women both in this region and elsewhere in the country.
